# Assessment of the proportion of neonates and children in low and middle income countries with access to a healthcare facility: A systematic review

**DOI:** 10.1186/1756-0500-4-536

**Published:** 2011-12-13

**Authors:** Aruna Chandran, Hadley K Herbert, Anne CC Lee, Igor Rudan, Abdullah H Baqui

**Affiliations:** 1 Departments of International Health and Pediatrics, Johns Hopkins University, 615 N. Wolfe Street, Suite E8622, Baltimore, MD 21205; USA; 2 Department of International Health, Johns Hopkins Bloomberg School of Public Health, 615 N. Wolfe Street, Baltimore, MD 21205; USA; 3 Centre for Population Health Research and Global Health Academy, The University of Edinburgh Medical School, Teviot Place, Edinburgh, EH8 9AG, Scotland, UK; 4 Department of International Health, Johns Hopkins Bloomberg School of Public Health, 615 N. Wolfe Street, Suite E8138, Baltimore, MD 21205; USA

## Abstract

**Background:**

Comprehensive antenatal, perinatal and early postnatal care has the potential to significantly reduce the 3.58 million neonatal deaths that occur annually worldwide. This paper systematically reviews data on the proportion of neonates and children < 5 years of age that have access to health facilities in low and middle income countries. Gaps in available data by WHO region are identified, and an agenda for future research and advocacy is proposed.

**Methods:**

For this paper, "utilization" was used as a proxy for "access" to a healthcare facility, and the term "facility" was used for any clinic or hospital outside of a person's home staffed by a "medical professional". A systematic literature search was conducted for published studies of children up to 5 years of age that included the neonatal age group with an illness or illness symptoms in which health facility utilization was quantified. In addition, information from available Demographic and Health Surveys (DHS) was extracted.

**Results:**

The initial broad search yielded 2,239 articles, of which 14 presented relevant data. From the community-based neonatal studies conducted in the Southeast Asia region with the goal of enhancing care-seeking for neonates with sepsis, the 10-48% of sick neonates in the studies' control arms utilized a healthcare facility. Data from cross-sectional surveys involving young children indicate that 12 to 86% utilizing healthcare facilities when sick. From the DHS surveys, a global median of 58.1% of infants < 6 months were taken to a facility for symptoms of ARI.

**Conclusions:**

There is a scarcity of data regarding the access to facility-based care for sick neonates/young children in many areas of the world; it was not possible to generalize an overall number of neonates or young children that utilize a healthcare facility when showing signs and symptoms of illness. The estimate ranges were broad, and there was a paucity of data from some regions. It is imperative that researchers, advocates, and policy makers join together to better understand the factors affecting health care utilization/access for newborns in different settings and what the barriers are that prevent children from being taken to a facility in a timely manner.

## Background

According to the World Health Organization (WHO), nearly 40% of all under-5 deaths in 2000 occurred in infants in their first 28 days of life [1]. The vast majority of these deaths (99%) occurred in low and middle income countries, where access to healthcare services is limited and/or lacking in quality [2]. In order to achieve the 4th Millennium Development Goal of reducing under-5 mortality by two-thirds between 1990 and 2015, the 3.58 million neonatal deaths that occur each year must be reduced [3].

Among neonatal deaths, 75% occur in the first week of life, with 25-45% occurring within the first 24 hours after birth. The majority of these deaths occur in the home [2]. Comprehensive antenatal, perinatal and early postnatal care has the potential to significantly reduce neonatal mortality.

In order to understand the factors contributing the maternal mortality, a model has been proposed by Thaddeus and Maine in which the three phases of delay in a woman receiving care during childbirth are laid out as follows: 1) Delay in seeking care on the part of the individual/family; 2) Delay in reaching an adequate healthcare facility; and 3) Delay in receiving adequate care at that facility [4]. According to these authors, these delays are inter-related, but are not inextricably linked, such that a delay in any one of these phases can result in an adverse outcome.

Adapting this model for neonatal mortality, the second phase of delay (delay in reaching an adequate healthcare facility) presents a formidable challenge particularly in low/middle income countries where access to health care facilities can be limited. Factors contributing to limited access to a facility include but are not limited to the distance to a facility, the costs of reaching and receiving care, lack of available roads/transportation, time away from household and childcare responsibilities, security concerns for reaching the facility, the time needed to reach and receive care, and cultural beliefs regarding taking a baby for care.

In order to understand how this second delay can best be addressed, it is critical first to quantify the level of access that neonates have to an adequate healthcare facility. This paper systematically reviews published data as well as DHS surveys to quantify newborn access to health facilities in low and middle income countries by extracting evidence of utilization of healthcare facilities by ill neonates. Because of the paucity of available data on access for newborns only, we expanded our search to include children < 5 years of age. We divide the information gathered from low and middle income countries by WHO region in order to make the information comparable to most published studies and to the data regularly gathered/published by the WHO. Gaps in available data by WHO region are identified, highlighting the need for future research and advocacy.

## Methods

### Definitions

The goal of this analysis was to conduct a systematic review of the available published literature that quantified what proportion of neonates have access to a healthcare facility in each WHO world region. However, due to the paucity of information and the wide range of definitions that were needed to be used, we focused on a systematic review of published literature, and supplemented this with information from the Demographic and Health Surveys (DHS) data. The context of this review was to inform a model of the potential impact of newborn lives saved by the development of novel diagnostics for neonatal infections, funded by the Bill and Melinda Gates foundation. For the purposes of this review, the following definitions were used.

#### Access

RAND defines access to healthcare facilities as "the ease with which a patient can gain entry to or utilize health care in the face of financial, geographical, organizational, cultural, and emotional barriers" [5]. Even in this definition as well as in the majority of publications, the concepts of "access to healthcare" and "utilization of healthcare" are used interchangeably. These concepts are fundamentally different, in that a person may not use a service even if they have access to it. Access (often labeled as "potential access") is usually thought of as when a disadvantaged individual lives in a place at a time when a capable healthcare delivery system is available. Quantifying access, which would essentially entail quantifying the range of available facilities and the barriers preventing people from utilizing them, is for obvious reasons very difficult. Therefore, utilization of healthcare services is often the only available proxy for access to a facility [6]. This is thought of as "realized" or "actual" access, which is when all barriers are removed and a sick individual actually presents to a healthcare facility. Therefore, for this paper, quantification of "utilization" of a healthcare facility is used to represent "realized access" and is used as the best available proxy for "access" to a healthcare facility.

#### Facility

A healthcare facility can vary in terms of the capabilities and infrastructure available. The level of available infrastructure is particularly important in the case of caring for sick neonates/young children, as adequate care often hinges on the availability of proper diagnostic/laboratory facilities as well as capability to deliver intravenous (IV) antibiotics. For the purposes of this paper, in order to present as much of the available data as possible, the term "facility" was used to refer to any permanent clinic or hospital outside of a person's home in which a "medical professional" (a provider not termed as a "traditional healer", "village doctor", "pharmacist", or non-trained person) was available.

### Search Strategy

A systematic literature search was conducted between March and July 2010 using PubMed/Medline, Embase, and the Cochrane Library. Search terms were based on the key words and mesh headings for terms representing the following: 1) "Infection; sepsis"; "Infant; neonate" to identify articles focusing on neonates/young children with serious illnesses that would warrant facility access/utilization; 2) A detailed listing of "Low and middle income countries" including other ways to express this category and the names of the countries themselves; and 3) "Access to health care" or "Health services utilization" terms representing different types of healthcare facilities, public/private sectors, and common barriers of use.

Articles in English, Spanish, French, or Portuguese were identified and de-duplicated. A single reviewer then screened articles based on the title/abstract for relevance, eliminating articles that clearly did not include the appropriate age group, geographic area, or topic area of access to care. This resulted in 117 articles which were reviewed by two reviewers; papers with original data presented were identified. A total of 14 articles were included in the final analysis.

The initial strategy was to focus only on data involving the neonatal (< 1 month of age) age group. However, due to the paucity of data available for this age group, the inclusion criteria were expanded in a second search to include studies focusing on children up to 5 years of age as long as the neonatal age group was not specifically excluded. Because utilization was used as a proxy for access, our focus was on neonates/young children exhibiting illness symptoms in order to quantify utilization of care at a healthcare facility. Data was extracted for children labeled as "sick", with more specific terminology including "suspected sepsis" or "severe illness", or with specific diagnoses such as "diarrhea" or "pneumonia". Data was included for those children who were taken a to a healthcare facility (outside of a person's home) staffed by a "medical provider" not labeled as a "traditional healer", "village doctor", "pharmacist", or "non-trained person".

#### Study Quality

The Child Health Epidemiology Group (CHERG) suggests criteria to evaluate the quality of studies that are focused towards the evaluation of interventional clinical trials [7]. In contrast, there has been no agreed upon ideal methodology for the evaluation of healthcare access or utilization in real-world populations. In this article, certain study components are presented to allow readers to judge study quality and representativeness relevant to our aims; however, we did not exclude any articles based on quality concerns. The population size (N) relevant to our aims (not necessarily the full enrollment sample in the study) is given. The design describes whether data was prospectively or retrospective collected, and the extent to which the study sample was representative of the general population. Facility infrastructure describes the precision with which the study defines "healthcare facility" in terms of quality/level of providers and diagnostic/laboratory/treatment services available. The denominator description focuses on the precision with which "illness" was defined/described, and the age range for which specific data was made available.

#### Demographic and Health Surveys (DHS) Data

To supplement the available data, information from available Demographic and Health Surveys (DHS) was extracted using statcompiler. DHS are nationally representative household surveys (usually representing 5,000 to 30,000 households) conducted in low/middle income countries to provide data on a range of indicators in the areas of health and nutrition. Data was extracted for children < 6 months of age (the lowest disaggregated age range provided in statcompiler) with symptoms of Acute Respiratory Infection that were taken to a health facility. The definition of ARI used by DHS was a mother's perception of a child who has cough, is breathing faster than usual with short, quick breaths or is having difficulty breathing, excluding children that had only a blocked nose.

## Results

**Figure 1:**
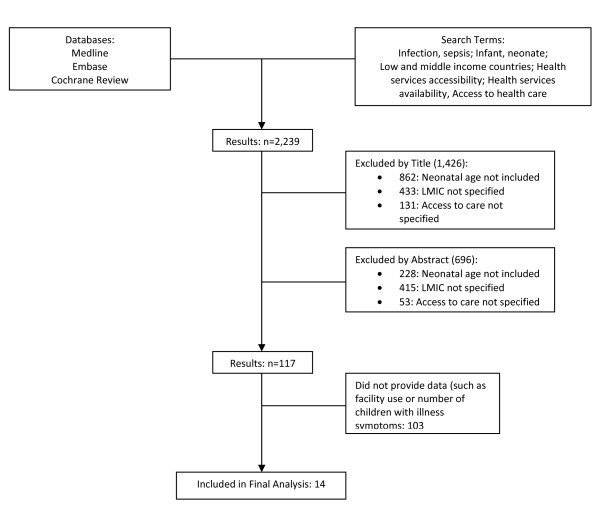
**Flow chart of systematic literature search and review**.

The initial broad search yielded 2,239 articles. Screening by title/abstract resulted in 117 articles, of which 14 presented data relevant for our aim (Figure 1).

Six clinical trials focused specifically on neonates and reported the proportion of children accessing health facilities in each study arm. Given that these trial interventions included behavior change to increase careseeking, data is reported here for only the control/non-intervention arm, in an attempt to capture the baseline healthcare utilization rates in these communities (Table 1). The studies were all randomized trials conducted in South Asia, encompassing WHO's South East Asia Region (SEAR), potentially limiting their generalizability to other parts of the world. The aims of these community-based studies included birth preparedness, education of danger signs and symptoms in the newborn, and targeted enhancing newborn care-seeking for illness. In these studies, the control clusters did benefit from improvements in equipment and training for the existing healthcare infrastructure, which may have reduced the 3^rd ^delay and increased care-seeking. The Awasthi study had an 80% facility utilization rate; however, study enrollment occurred at the facility so it would be expected that a high proportion of these individuals would return to the facilities for care [8]. In the community-based studies, the range of proportions of sick neonates who utilized a healthcare facility in the control arms was 10 to 48%.

**Table 1:** Proportion of Sick Neonates in Low and Middle Income Countries Accessing Care from a Healthcare Facility

Citation	Country (WHO Region)*	N	Facility Infrastructure Level	Prospective vs Retrospective	Population Based vs Sample	Denominator description	% Utilizing Facility
Awasthi, 2009 [8]	India (SEAR)	510 enrolled in baseline facility based survey	Public hospital with doctors and basic diagnostic and treatment facilities	Prospective	Sample: 2 urban public hospitals in Lucknow	Enrolled sick newborns who came to the facility	80% utilized the facilities in baseline assessment

Bari, 2006 [15]	Bangladesh (SEAR)	2,290 newborns in comparison sample	Private hospital with specialized pediatric care	Prospective	Sample: Mirzapur subdistrict, Tangail district	Sick newborns who utilized the local hospital	18-23% utilized referral hospital

Dongre 2009 [16]	India (SEAR)	503 mothers of babies < 12 months	Public or private hospitals attended by doctors	Prospective	Sample: Wardha district, Maharashtra	Newborns with clinical danger signs presenting to local hospitals	48% utilized local hospital in needs assessment phase

Kumar, 2008 [17]	India (SEAR)	1079 live births in "control" arm	Primary health centers with trained physicians	Prospective	Sample: Shivgarh district, Uttar Pradesh	296 newborns with "any" illness	16.7% were attended by a doctor or nurse/midwife

Manandhar, 2004 [18]	Nepal (SEAR)	3,226 live births in control areas	Not specified	Prospective	Sample: Makwanpur district	Neonates with signs of illness (cough, fever, diarrhea)	10% utilized a health facility for illness

McPherson, 2006 [19]	Nepal (SEAR)	Number in baseline survey population not specified	Not specified	Household survey before/after community intervention	Sample: Siraha District	Not specified	11-17% utilized postnatal care services

* SEAR: South East Asia Region

Table 2 shows the data from studies including infants/young children. The lowest age group for which data was available is presented. The majority of these were cross-sectional household surveys. They were population-based; however, all were done in representative samples from a particular area or district within the country and therefore may or may not be nationally generalizable. The vast majority used a very open definition of "healthcare facility" which ranged from individual clinics to tertiary care hospitals. For the majority, it was not possible to discern the level of infrastructure available at the various health facilities. In addition, there was a wide range of illness definitions, ranging from children with mild upper respiratory infection symptoms to verbal autopsy studies of children who had passed away.

**Table 2:** Proportion of Sick Children in Low and Middle Income Countries Accessing Care from a Healthcare Facility

PI	Country (WHO Region)	N	Design	Facility Infrastructure	Population Based vs Sample	Denominator	% Utilization
Amarasiri de Silva, 2001 [20]	Sri Lanka (SEAR)	174	Cross sectional survey	Varied: Includes private doctor or hospital, public or private	Population based: Kurunegala district	< 12 mos, children with ARI or diarrhea	69.9% with ARI, 86.8% with diarrhea

Andy, 1990 [21]	Fiji (WPR)	43	Cross sectional survey	Primary health center staffed by nurses	Population based: Cicia Island	0-4 yrs, reported illness symptoms	40% utilized health center

Armstrong, 2008 [22]	Tanzania (AFR)	674	Cross sectional household survey	Varied: "Western style provider", includes hospital, health center, and dispensaries	Population based: Five districts, Southern Tanzania	< 23 months, sick in last 2 weeks	46% utilized outside facility

Feikin, 2009 [23]	Kenya (AFR)	4,881	Cross sectional survey	Peripheral health facilities (DSS clinic)	Population based: Asembo	< 1 year, sick children	23% visited the clinic

Hadad, 2002 [24]	Brazil (AMR)	395	Verbal autopsy survey	Tertiary care hospital	Sample: Belo Horizonte	< 1 yr, had been taken to a hospital prior to death	91% visited a hospital

Kakai, 2009 [25]	Kenya (AFR)	242	Cross sectional survey	Government run health center	Sample, Bokoli, Bungoma East District	< 5 yrs, maternal diagnosis of malaria	28.1% utilized health facility

Pandey, 2002 [26]	India (SEAR)	790	Prospective cohort	Outside facility of a allopathic or homeopathic provider	Sample: Four villages near Kolkata	< 5 years, sick children taken to qualified professional	12.9% utilized facility

Yount, 2004 [27]	Egypt (EMR)	3,125	Longitudinal household survey study	Government hospital or clinic	Sample: Minya governorate	< 1 yr, sick children	12% utilized facility

*AFR: African Region, AMR: American Region, EMR: Eastern Mediterranean Region, SEAR: South East Asia Region, WPR: Western Pacific Region

Unfortunately, there were no studies from the European (EUR) region that fit our criteria. Two significant outliers in terms of proportion of sick children utilizing a healthcare facility were Brazil (91%) and Sri Lanka (69.9 - 86.8%). Notably, the study in Brazil was a retrospective analysis of children who subsequently died, so it would not be unexpected that the majority of those would have been taken to a facility. Data from the rest of the studies indicates a broad range of 12 to 86% utilizing healthcare facilities when sick.

**Figure 2:**
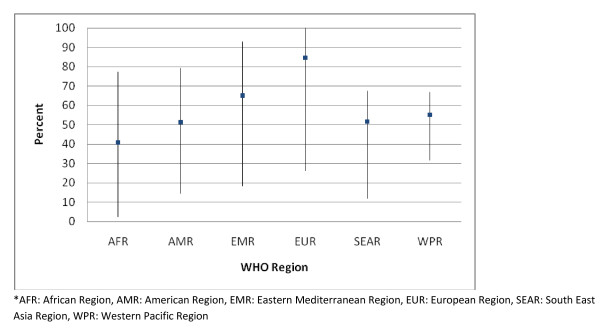
**Demographic and Health Surveys: proportion of infants < 6 months in low and middle income countries with ARI symptoms taken to a healthcare facility; Median with Ranges**.

A summary of the data extracted from DHS surveys is presented in Figure 2. A global median of 58.1% of infants < 6 months were taken to a facility for symptoms of ARI. The highest median (84.7%) came from EUR, with some countries (Azerbaijan, Turkmenistan, Moldova, and Uzbekistan) reporting that 100% of sick children were taken to a health facility. The lowest median (40.9%) was from AFR, with the 2004 survey in Chad reporting that only 2.3% of children with symptoms of ARI were taken to a facility.

## Conclusions

Due the divergent sources from which data was extracted, it is not possible to generalize an overall number of neonates or young children that utilize a healthcare facility when showing signs and symptoms of illness. From published neonatal intervention trials aimed to improve birth preparedness and care-seeking practices, it appears that in the SEAR region, a range of 10-48% of neonates utilize a health facility when ill. In the same region, 69-87% of children < 1 year of age in Sri Lanka were taken to a health facility for ARI or diarrhea. In contrast, in AFR and EMR, 12-46% of children < 1 year of age utilized a healthcare facility when ill. Broadening the age range to encompass children < 5 years of age, 12.9-40% of children (in SEAR, AFR, and WPR) attended a healthcare facility for illness. The study from AMR was retrospective focusing on children who had passed away; 91% of those had been taken to a healthcare facility for illness symptoms. Unfortunately there were no studies found from the EUR region that presented data relevant to healthcare facility utilization. The summary of available DHS surveys shows that 58.1% of infants < 6 months of age are taken to a healthcare facility for symptoms of ARI. Clearly, low utilization of healthcare facilities is an important component of the high rates neonatal mortality that persist in low and middle income countries.

This analysis had several limitations. First, utilization was used as a proxy for access. There are interesting and important studies attempting to identify the barriers to access to care at a healthcare facility and therefore assess access instead of utilization [9,10]. For this paper, in which we attempted to quantify access to care at a healthcare facility, we focused on evidence of facility-based care utilization. This then necessitated focusing on sick neonates/young children that utilized a healthcare facility instead of allowing a focus on all neonates/young children that might have access to a healthcare facility when needed. Secondly, due to logistical constraints, articles written in non-Latin alphabet (i.e., Russian, Chinese, etc.) were not included in this analysis.

Importantly, it was not always possible to determine the level of infrastructure available in a healthcare facility mentioned in a study. Olmsted *et al*. defined level of facility as having no, minimal, modest, or advanced infrastructure. These are defined as follows: 1) No infrastructure: care at home or in the community, with no staff expertise or equipment available; 2) Minimal infrastructure: a community clinic or room, staffed by a person with minimal expertise (usually includes traditional healers or trained community health workers), and with no laboratory or intravenous medicines; 3) Moderate infrastructure: a community hospital or urban health clinic staffed by nurses or ancillary providers with poor laboratory conditions and only basic intravenous medicines available; and 4) Advanced infrastructure: a hospital with full laboratory capabilities and access to all recommended antibiotics/medicines [5,11]. Per Western standards of care for treating suspected sepsis in a neonate, advanced infrastructure is essential (including invasive procedures, laboratory facilities, and ability to give IV antibiotics). In contrast, studies from developing countries have shown significant impact on neonatal mortality using community-level interventions [12-14]. Further studies are needed to understand exactly what type of facility access would be needed in order to treat neonatal sepsis successfully. Because our analysis focused evidence of healthcare facility utilization by ill neonates and young children, we chose to include all studies mentioning facility utilization regardless of the level of infrastructure available or mentioned.

Despite the limitations of this analysis, the clearest lesson learned from this analysis is that there is a scarcity of data regarding the access/availability of facility-based care for neonates in many areas of the world. Our paper highlights the generally low to mid level of utilization of healthcare by sick neonates/young children in most low and middle income countries. Further evaluations are needed to understand factors affecting health care utilization for sick newborns in different settings and what are the barriers that prevent these neonates from being taken to a facility in a timely manner. Commonly and widely utilized survey tools such as the DHS surveys could be expanded to include questions related to perceived barriers on accessing facilities in the critical window in which a sick neonate must receive care. It is important to put appropriate health systems infrastructure in place to allow for a sick neonate to be recognized at any level of health facility so that those children at highest risk can be promptly referred for appropriate care and treated. This may involve training of community health workers or local providers, or the development of a point-of-care diagnostic test that is non-invasive and inexpensive to administer.

A recent meta-analysis of community-based intervention trials showed that home-based neonatal care can reduce neonatal mortality by 38% (Relative Risk 0.62; 95% CI: 0.44, 0.87) [12]. To further reduce the neonatal mortality burden, prompt access to appropriate healthcare facilities is critical. Without increasing access to healthcare facilities, thereby diminishing this important delay in the appropriate care of a neonate who becomes ill, further progress cannot be made in decreasing the global burden of neonatal mortality. It is imperative that researchers, advocates, and policy makers join together to better understand and combat this important problem.

## Competing interests

The authors declare that they have no competing interests.

## Authors' contributions

AC, HH, and AL participated in conducting the searches, reviewing articles, and extracting the data. IR and AB provided technical guidance and support. All authors were involved in designing the study and drafting the manuscript. All authors read and approved the final manuscript.
